# Melatonin and Cancer: A Polyhedral Network Where the Source Matters

**DOI:** 10.3390/antiox10020210

**Published:** 2021-02-01

**Authors:** Maria-Angeles Bonmati-Carrion, Antonia Tomas-Loba

**Affiliations:** 1 Chronobiology Laboratory, Department of Physiology, IMIB-Arrixaca, University of Murcia, 30100 Murcia, Spain; 2 Ciber Fragilidad y Envejecimiento Saludable, 28090 Madrid, Spain; 3 Circadian Rhythm and Cancer Laboratory, Department of Physiology, IMIB-Arrixaca, University of Murcia, 30120 Murcia, Spain

**Keywords:** melatonin, cancer, antitumor, antioxidant, circadian, chronobiotic, pineal, extrapineal, immunomodulatory, light at night

## Abstract

Melatonin is one of the most phylogenetically conserved signals in biology. Although its original function was probably related to its antioxidant capacity, this indoleamine has been “adopted” by multicellular organisms as the “darkness signal” when secreted in a circadian manner and is acutely suppressed by light at night by the pineal gland. However, melatonin is also produced by other tissues, which constitute its extrapineal sources. Apart from its undisputed chronobiotic function, melatonin exerts antioxidant, immunomodulatory, pro-apoptotic, antiproliferative, and anti-angiogenic effects, with all these properties making it a powerful antitumor agent. Indeed, this activity has been demonstrated to be mediated by interfering with various cancer hallmarks, and different epidemiological studies have also linked light at night (melatonin suppression) with a higher incidence of different types of cancer. In 2007, the World Health Organization classified night shift work as a probable carcinogen due to circadian disruption, where melatonin plays a central role. Our aim is to review, from a global perspective, the role of melatonin both from pineal and extrapineal origin, as well as their possible interplay, as an intrinsic factor in the incidence, development, and progression of cancer. Particular emphasis will be placed not only on those mechanisms related to melatonin’s antioxidant nature but also on the recently described novel roles of melatonin in microbiota and epigenetic regulation.

## 1. Introduction

Melatonin, a derivative of tryptophan, is an indoleamine (N-acetyl-5-methoxytryptamine) that was originally isolated from beef pineal gland by Lerner et al. in 1958 [1]. The first studies on melatonin were focused on its chronobiotic function, as it is one of the main signals in the circadian system. In higher organisms, this hormone is rhythmically secreted by the pineal gland and it has been adopted as a darkness molecule [2] due to its higher plasma concentrations during the night and its acute suppression by light at night (LAN).

However, apart from the central role in circadian synchronization and sleep promotion in humans exerted by melatonin produced by the pineal gland, melatonin is a versatile and ubiquitous molecule, also produced by extrapineal peripheral tissues, that exerts a wide variety of functions in the organism. Among them, its antioxidant activity has been widely documented, revealing most of the required characteristics of an efficient antioxidant [3]. Indeed, melatonin appeared early in evolution, and this was presumably its original function in unicellular organisms exposed to highly oxidant environments, especially during the daytime [4]. Melatonin has also been described as an anti-inflammatory, immunomodulatory, pro-apoptotic, antiproliferative, and anti-angiogenic factor, properties that make it a powerful antitumor agent, as well as a modulator of reproduction in vertebrates. 

Although for several years after its isolation melatonin was considered just a pineal hormone with circadian and circannual functions, there is increasing evidence of local melatonin production in many extrapineal tissues. Among the extrapineal melatonin sources are the gastrointestinal and reproductive tract, retina, brain, lens, cochlea, immune system, and skin, which are all reviewed by Acuña-Castroviejo et al. [5]. Thus, whereas chronobiotic function is mainly exerted by melatonin secreted by the pineal gland in a rhythmic manner, extrapineal melatonin has been described to produce local effects, some of them closely related to its antioxidant activity. However, to the date how extrapineal melatonin production is regulated and whether pineal melatonin has any role in this regulation is not entirely understood and may constitute an important issue to unravel.

Epidemiological studies that show a correlation between light at night (or inadequate light exposure patterns) and cancer prevalence suggest that melatonin secreted by the pineal gland has an important role in cancer prevention, initially attributed to its role in circadian synchronization. Additionally, in the context of the direct antitumor effects of melatonin that have been widely documented both in vitro and in vivo, local extrapineal melatonin may also have an important role. However, as stated before, there is not much information about how extrapineal melatonin is regulated and whether the circadian system in general and pineal melatonin in particular play a role in this regulation. Here, we have revised the existing evidence to open the way to further research on this central issue, as well as on how the environment and circadian/sleep/nutrition habits may eventually affect this extrapineal production, which is a topic of interest in terms of cancer prevention.

The antitumor actions of melatonin on different types of cancers (especially those that are hormone-dependent) have been widely studied and will be reviewed in this article, including melatonin’s effect on proliferation, immune response and inflammation, replicative immortality, angiogenesis, metastasis, genome instability, and cell death, with special emphasis on the antioxidant properties taking part in these processes. One effect related to its antioxidant actions that has been recently gaining attention is its participation in the regulation of antioxidant and pro-inflammatory genes via epigenetic on/off mechanisms [6]. 

In addition, there is increasing evidence of the role of melatonin in controlling the microbiota composition [7] and suppressing pathogenic bacteria in the intestine [8], some of these processes mediated by its antioxidant activity. The microbiota of the gastrointestinal tract has an important role in human physiology and metabolism [9], and considering its implication in the pathogenesis of different cancers [10] we believe that the melatonin–microbiota–cancer link should be more extensively explored. To facilitate this purpose, in this review article we revise the existing evidence on the melatonin–microbiota and microbiota–cancer links.

Melatonin should be considered as a molecule that acts at different levels; regulates a wide variety of biological functions, with multiple sources and targets; and is subject to different regulatory processes. How pineal melatonin could regulate all these processes in a direct or indirect way, including the regulation of extrapineal melatonin and its functions, is an aspect of great interest that remains to be unraveled. The possible effect of the physiological environment on health, promoted by different habits (sleep, circadian, or nutritional), means that this molecule could be the possible link between a healthy lifestyle and cancer prevention.

## 2. Melatonin: A Ubiquitous and Conserved Molecule through Evolution

Although primarily discovered in mammals, there is evidence that suggests melatonin appeared early in evolution. Almost three decades after its isolation, the dinoflagellate *Lingulodinium polyedrum*, a unicellular organism, was described to have the capacity to synthesize melatonin [11]. After that first evidence of the indoleamine in unicellular organisms, melatonin has been identified in many and distant taxa, including bacteria [12,13], trypanosomids [14], euglenoids, alveolates, pheophyceans, rhodophyceans [15], unicellular chlorophyceans [13], plants [16,17], fungi [13,18,19], and members of all major clades of invertebrates [15], as reviewed in [4]. This omnipresence in virtually all the clades suggests that melatonin appeared early in evolution and provided organisms with an adaptative advantage.

When, approximately 3.2 billion years ago, cyanobacteria integrated photosynthesis as a fundamental part of their metabolism, they started to deliver oxygen that was initially captured by iron and other organic matter with no impact on the reducing atmosphere. However, around 2.4 billion years ago oceans became saturated and atmospheric oxygen began to rise, eventually provoking the Great Oxygen Event. Free oxygen in the atmosphere became a challenge to the survival of existing organisms and was responsible for the first mass extinction on Earth [20]. Only those cyanobacteria that were able to produce molecules to scavenge oxygen and nitrogen free radicals (ROS and NOS) were able to survive that catastrophe. Indeed, melatonin likely played an essential role those days, effectively protecting organisms from the oxidative stress at the beginning of that oxidant environment [21], especially during the daytime.

**Figure 1:**
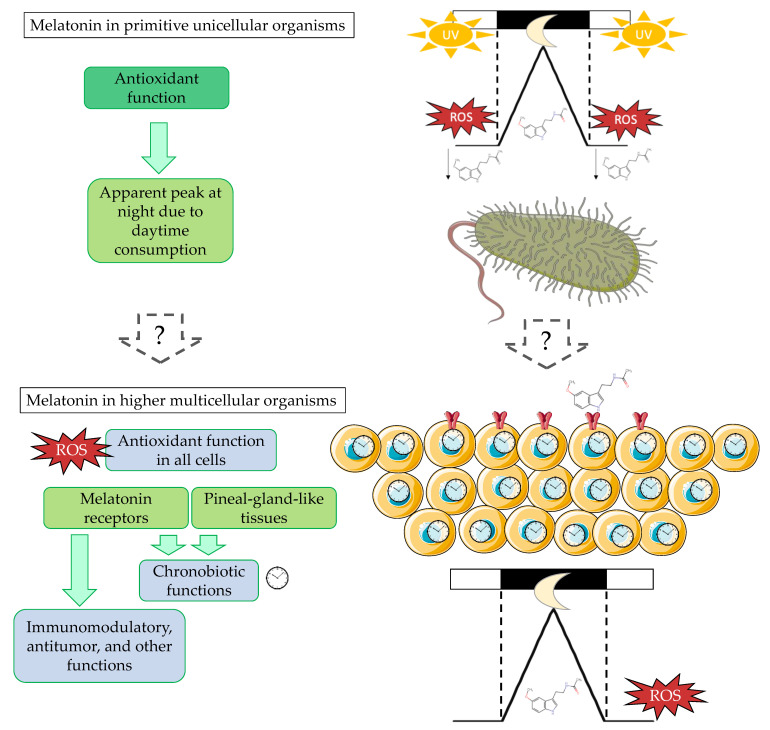
Melatonin’s biological functions throughout evolution. In primitive unicellular organisms (**upper panel**), its antioxidant primary function generated an apparent concentration peak at night. Subsequent events drove the appearance of melatonin receptors and pineal gland-like tissues (**lower panel**), permitting higher multicellular organisms to adopt melatonin as a chronobiotic (darkness) signal while maintaining local extrapineal melatonin production in most organs and tissues, exerting its antioxidant original function. Melatonin in multicellular organisms also presents a wide range of actions, such as immunomodulatory and antitumor, among others. This figure was built with SMART resources (Servier Medical Art) and licensed under a Creative Common Attribution 3.0 Generic License. See http://smart.servier.com/.

Indeed, compared to the scotophase (dark phase), greater amounts of melatonin were consumed during the photophase (light phase) due to the ultraviolet irradiation and the large quantities of free radicals generated during the photoshynthesis [22]. This led to an apparent melatonin rhythm in a light/dark cycle with a peak during the scotophase and lower concentrations during the photophase (Figure 1, upper panel). This process has been observed in dinoflagellate extracts [23] and has been hypothesized to be the origin of the potential of melatonin to become the chemical darkness signal [24]. 

Thus, the ability to synthesize melatonin was retained in eukariotes probably through the process of endosymbiosis, as hypothesized by Manchester and colleagues [21]. Indeed, these authors also suggest that current mitochondria have retained the ability to synthesize melatonin from these original organisms. Although the mitochondrial production of melatonin has been demonstrated, great efforts are required to fully understand how it is coordinated with its production in the cytoplasm. However, melatonin is able to preserve the integrity of mitochondria, helping to maintain cell functions and survival. It is not surprising that, in a highly oxidant environment such as in mitochondria, melatonin exerts essential functions, such as directly scavenging ROS and NOS; stimulating antioxidative enzymes; increasing the efficiency of the electron transport chain, thereby limiting electron leakage and free radical generation; and promoting ATP synthesis [25]. 

In higher organisms, the melatonin structure remains unchanged and has retained its primary antioxidant function while gaining a number of other critical roles, such as the modulation of seasonal reproduction; anti-inflammatory and immunomodulation activities (as reviewed in [4]); and, of primary importance, circadian synchronization (and sleep promotion in diurnal animals) [21] (Figure 1, lower panel). Indeed, when multicellular organisms appeared, coordination between cells and with external cyclic environment became a basic aspect to work on. Thus, oxidative stress, external cyclic changes, and the higher complexity of organisms drove them to “invent” a way to solve all these challenges with one simple molecule: melatonin. Its function as a darkness signal in multicellular organisms was likely mediated by membrane receptors [26], whose appearance permitted melatonin to acquire many other functions while keeping its initial structure and antioxidant function.

In multicellular organisms, most cells have preserved their capacity to locally produce high amounts of melatonin with, presumably, mainly antioxidant, tissue factor, autocoid, and paracoid functions [27]. However, at some point, melatonin receptors and pineal gland or pineal gland-like tissues evolved from neurons to produce a receptor-mediated chemical signal that “informed” the rest of the cells about the light–dark cycle the organism was exposed to [28]. Thus, interestingly, evolution kept the same molecule that had been useful for antioxidant purposes and developed the needed structures to make good use of its potential as a chronobiotic.

## 3. Biology

Melatonin (N-acetyl-5-methoxytryptamine) is a derivative of tryptophan and it is biochemically classified as an indoleamine. This molecule can exert its multiple functions directly or through receptors, as will be discussed below. So far, three melatonin receptors have been described: MT1-3. MT1 and MT2 are considered G-protein-coupled membrane receptors, while MT3 is a cytosolic enzyme quinone reductase 2 that presents strong antioxidant actions when bound to melatonin. Besides this, melatonin can interact with intracellular proteins such as calmodulin, tubulin, and p300, among others; transcription factors such as NF-κB or FOXO3; as well as nuclear receptors—e.g., RZR/RORs and ERα. Thus, these factors may potentiate the antitumor actions of melatonin [29].

### 3.1. Regulation, Synthesis and Secretion

#### 3.1.1. Pineal Melatonin

Tryptophan is the first precursor of melatonin in vertebrates and is hydroxylated by the enzyme tryptophan hydroxylase, becoming 5-hydroxytryptophan. The second step consists of decarboxylation, which transform 5-hydroxytryptophan into serotonin (5-hydroxytryptamine). Then, two key enzymes (arylalkylamine N-acetyl transferase, AANAT; acetyl-serotonin O-methyltransferase, ASMT) subsequently convert serotonin to melatonin in two steps. 

How is this production regulated in the pineal gland? The suprachiasmatic nuclei, the central pacemaker in the circadian system (see Section 4.2), is entrained by light via the retino–hypothalamic pathway and generates a circadian rhythm. This is the signal that passes via the paraventricular nucleus (although with some controversy), hindbrain, spinal cord, and superior cervical ganglion, and reaches pinealocytes at the level of membrane noradrenergic receptors (although astrocytes and microglia have also been reported to produce melatonin [30,31]). At the molecular level, during the subjective night noradrenaline binds with G-protein-coupled α and β-adrenergic receptors, increasing the intra-cellular levels of cAMP (cyclic adenosine monophosphate) in pinealocytes that activate protein kinase A II (PKA II), which phosphorylates cAMP response element binding protein (CREB), eventually inducing the expression of the key enzyme, AANAT. In primates, the rhythm seems to be generated through the phosphorylation of AANAT [32], this enzyme being generally considered the rate-limiting enzyme in melatonin synthesis, although acetylserotonin-O-methyltransferase (ASMT) has been also considered as the enzyme that limits the synthesis of the indoleamine [33]. After the described noradrenergic stimulation produced during the dark phase, AANAT can increase 30–70-fold, thus producing a peak of melatonin secretion [2].

The subcellular fraction where melatonin has been suggested to be synthesized is the mitochondria [34,35]. Indeed, pinealocytes contain a great amount of mitochondria [36] and, interestingly, the relative volumes of this organelle exhibit a circadian rhythm in pinealocytes, with greater volumes during the dark period compared to the daytime, consistent with the melatonin synthetic peak [37]. Melatonin also seems to be associated with dynamic alterations in mitochondria which are mediated by fission, fusion, and mitophagy activities (reviewed in [36]).

The SCN is thus in control of pineal melatonin formation and secretion, in close relation to the light–dark information received through the retino-hypothalamic pathway. Although the oscillation of the rate-limiting AANAT is mainly driven by the SCN, the pineal gland also possesses its own clock, which seems to entail the periodic facilitation of responsiveness rather than the autonomous generation of the melatonin rhythm. Apart from the SCN intrinsic oscillation entrained by the light–dark cycle, pineal melatonin is also acutely suppressed by light at night (LAN) [38,39,40,41,42,43,44], this process being independent of the phase-shifting effects. This photic shut-off is particularly pronounced if the spectral composition is in the range of 460–480 nm, matching the melanopsin (photopigment responsible for the photosensitivity of intrinsically photosensitive retinal ganglion cells, ipRGC) peak of sensitivity. The photic shutoff causes a rapid suppression of melatonin biosynthesis and release. 

Thanks to the profuse vascularization of the pineal gland, melatonin is rapidly secreted into the bloodstream and spinal fluid after its biosynthesis [45], where it is transported free or bound to albumin [46]. Melatonin reaches levels of 0.5–1 nM during the night in plasma, a concentration that should be considered when designing experimental protocols to address the actions of physiological pineal melatonin. It has a relatively short half-life, since it is quickly metabolized in the liver to 6-sulfatoxy melatonin (aMT6s, metabolite commonly used to infer melatonin rhythm), which is eventually excreted in urine [2]. 

#### 3.1.2. Extrapineal Melatonin

During the first years after its discovery, melatonin was considered a pineal hormone with chronobiotic functions. However, in the late 1960s and early 1970s, evidence of extrapineal melatonin synthesis was found in the habenula, the Harderian gland, and the retina [47]. After these first pieces of evidence, multiple studies have demonstrated the synthesis of melatonin in other organs/tissues than the pineal gland, such as the brain, immune system, gastrointestinal and reproductive tract, skin, lens, and cochlea. Although all these sources have been extensively reviewed by Acuña-Castroviejo et al. [5], we will focus here on extrapineal melatonin produced in the retina, immune system, and gastrointestinal tract, where the synthesis pathway is similar to that in the pineal, although differently regulated. 

##### Retina

Melatonin has been suggested to be locally synthesized by rods, cones, and retinal ganglion cells (RGCs) in a circadian manner [48,49,50], with an overall peak during the night in both nocturnal and diurnal rodents [50]. Melatonin rhythm presumably regulates dopamine secretion in amacrine cells [51,52] and eventually controls the alternation between light- and dark-adaptive effects [53,54]. Additionally, the retinal pigment epithelium (RPE) seems to constitute another source of melatonin in the eye, also expressing melatonin receptors (MT2, RORα1, RORα4, and quinone oxidoreductase (NQO2)) [55]. In humans, this rhythmic biosynthesis of melatonin in the retina seems to be mediated by the rhythmic post-transcriptional regulation of AANAT, with no required changes in gene expression [56].

Although in pinealectomized animals melatonin rhythm in the retina persists, which suggests there is an intrinsic rhythm in melatonin production, the peak concentration increases [57,58]. This suggest that, although the rhythm of local melatonin biosynthesis is autonomous in the retina, it may be also subjected to some type of regulation under the pineal melatonin. This double circadian regulation of melatonin in the retina, probably meant to ensure adaptive functioning, is not surprising, considering that the retina (namely, ipRGC) is the place where light information enters the circadian system. The retinal circadian clock has been previously widely documented and reviewed [59].

Moreover, melatonin locally produced in the retina also has an important function as an antioxidant—e.g., protecting the photoreceptor outer segment of membranes from photo-oxidative stress [60,61] and counteracting ischemic injury in RPE cells [62]. Interestingly, this antioxidant protecting effect of melatonin has been demonstrated both at physiological (10^−10^–10^−8^ M) and higher (10^−6^–10^−4^ M) concentrations through receptor-mediated or direct mechanisms, respectively [63].

Thus, melatonin produced by the retina seems to be essential for the functioning of the eye, the first step for light to enter the circadian system and, thus, is potentially essential for correct circadian synchronization. 

##### Immune System

Although pineal melatonin plays an important role in immunomodulation (which will be discussed later in relation to antitumor properties), different immune cells and tissues are also able to locally synthesize melatonin. Namely, melatonin has been found to be produced in bone marrow, spleen, thymus, lymphocytes, natural killer cells, eosinophilic leukocytes, mast and endothelial cells, and platelets [64,65,66,67]. 

In bone marrow, locally produced melatonin [68] likely exerts on-site protection against oxidative stress in these vulnerable hematopoietic cells, probably by enhancing the immune capacity of derived immune cells such as lymphocytes [69]. The rhythmic expression of melatonergic enzymes has been recently described in this tissue, with a peak during the night, which correlates with the intracellular concentration of melatonin [70]. These authors also support the idea that the bone marrow could rhythmically produce its own melatonin, complementing the one from the pineal gland, thus not discarding the possibility of circulating uptake.

In the spleen, although direct data on the function of local melatonin in this organ are limited, there is much evidence about melatonin’s effects on the activation and differentiation of T cells [71], as well as on the increase in lymphocytic proliferation [72,73,74]. Interestingly, melatonin and its biosynthetic enzymes decrease with age in the spleen, as well as the proliferation of lymphocytes [73,74], demonstrating a direct correlation between melatonin and the immune response of spleen cells. In addition, melatonin may play an important role in surveillance against the infection, inflammation, and recovery phases of acute defence response [75,76]. Regarding the variation in the melatonergic biosynthetic machinery, it has been recently described to present higher levels of expression during the diurnal phase, with the melatonin peak occurring in the dark phase and thus with no correlation between melatonin levels and the expression of its enzymes [70]. These authors suggest the possibility that nocturnal melatonin in the spleen is derived from the pineal gland, although the endogenous production of melatonin in the spleen has been also previously demonstrated [64].

Similarly, in the thymus of adult rats melatonin production seems to be synthesized under the control of pineal melatonin [77]. In human lymphocytes, great amounts of melatonin have been found, with values up to five times greater than the values of plasma concentrations at night [66,67]. In these cells, local melatonin production has been related to the intra-, auto-, and paracrine regulation of interleukin 2 (IL-2) and IL-2 receptor (IL-2R) [67], probably mediated by membrane and nuclear melatonin receptors. 

In activated macrophages, it has been suggested that local melatonin may exert protection from the damage induced by high levels of NO^●^ and/or the product, peroxynitrite, produced under certain conditions [5]. In mast cells, membrane melatonin receptors have been reported to be present, as well as the AANAT and ASMT machinery, although it is also likely that these cells can uptake extracellular pineal melatonin from plasma or their own melatonin via autocrine or paracrine mechanisms [78]. 

##### Gastrointestinal Tract

Melatonin is also locally biosynthesized by the gastrointestinal tract (GIT). It was first detected in the GIT of post-natal rats with no pineal production of melatonin, and subsequent studies have identified AANAT and ASMT activity in enterochromaffin cells. The melatonin levels found in the GIT are up to 10–100 or even 400-fold the levels found in serum and the pineal gland, respectively. However, whether the content is exclusively derived from local biosynthesis in enterochromaffin cells is still unclear. On one hand, it has been suggested that part of the gastrointestinal melatonin could be result from the food source used [79,80,81,82,83,84,85]. Indeed, foods’ melatonin content varies extensively [17,86,87,88,89], which may explain the species-dependent differences in the GIT melatonin concentrations regarding the food usually consumed [83,84,85]. Another suggested source of melatonin in the GIT is the microbiota [5], considering that microorganisms are able to produce melatonin in large amounts [4,12], which will be reviewed later in this article.

Whether the gastrointestinal biosynthesis of melatonin is rhythmic is not clear either. Some authors have found a temporal pattern of AANAT mRNA in young mice, which is consistent with the increase in fecal melatonin during the night. However, the same authors found AANAT mRNA during the day in the middle-aged and aged mice [90], which would suggest an age-dependent melatonin regulation. However, other studies have not found any diurnal pattern of melatonin biosynthesis in the GIT [5]. In humans, a transient post-prandial elevation of serum melatonin has been detected and related to the release of melatonin from the GIT. This has been linked to postprandial somnolence [85] and a decrease in the core body temperature [91,92], although it is not clear whether this slight increase in serum melatonin after a meal is sufficient to cause this effect. 

Among the possible functions of melatonin in the GIT, it has been suggested to participate in propulsive motility in intra-, auto-, and paracrine ways [93], causing opposite effects of those produced by serotonin [94,95,96]. Therefore, whereas serotonin exerts contractile effects, melatonin produces relaxation on the outer smooth muscle layer [97,98] through the MT2 membrane receptor [94,99]. Melatonin in GIT also participates in the neutralization of acid content in the duodenum along with the MT2 receptors, stimulating the secretion of bicarbonate ions [100,101,102,103]. In the liver, the melatonin content has been found to be 15-fold higher than in serum [104] and hepatocytes also release melatonin into the bile, where it also reaches high concentrations [105,106]. Although local biosynthesis has been demonstrated by the presence of AANAT and ASMT mRNAs and their activities, some of the hepatic melatonin comes from the GIT via the hepatic portal vein [85,107,108]. Melatonin receptors are also expressed in the liver, which suggests the relevant functions of this molecule in this organ. Indeed, there is evidence for the inhibitory effects of melatonin on hepatic cancer [109,110].

#### 3.1.3. Possible Interplay between Pineal and Extrapineal Melatonin

**Figure 2:**
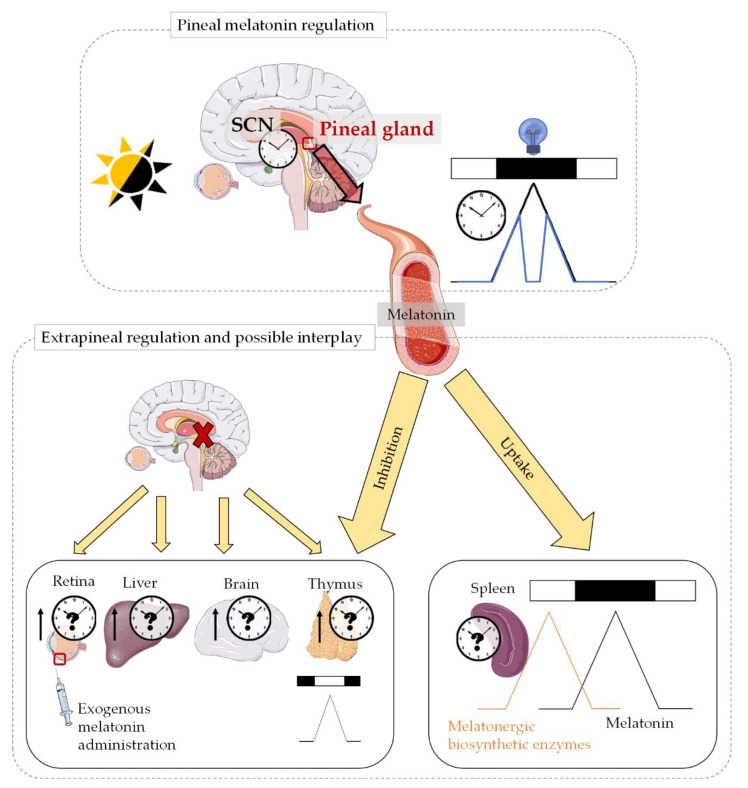
Regulation of pineal melatonin secretion (**top**) subjected to circadian rhythm originated in the suprachiasmatic nuclei (SCN), entrained by the light–dark cycle (peak at night), and also subjected to an acute suppression by light at night. Possible circadian regulation of extrapineal melatonin production (**bottom**) in the retina, liver, brain, thymus, and spleen. A possible interplay with pineal melatonin has been suggested (yellow arrows). Retina is able to uptake melatonin from exogenous administration, while pineal melatonin seems to inhibit its local production, since pinealectomy increases local melatonin levels as well as those in the liver, brain, and thymus. In addition, in the thymus the local melatonin content peaks during the day, which confirms the possible local inhibition by pineal melatonin. The spleen exhibits a peak of melatonin levels during the night, while the melatonergic biosynthetic enzymes are mainly expressed during the day, indicating a possible uptake of melatonin from circulation. Further studies are needed to fully understand this relationship. Cross on the pineal gland means pinealectomy. ↑ means increased local melatonin levels. This figure was built with SMART resources (Servier Medical Art), licensed under a Creative Common Attribution 3.0 Generic License. See http://smart.servier.com/.

Although pineal melatonin has received most of the attention in contrast to melatonin locally produced in extrapineal tissues, the latter may have a relevance in physiology and pathology that might not have been extensively understood yet. Thus, it is of great importance to deepen our knowledge of how extrapineal melatonin levels or production are regulated and whether pineal melatonin may be a signal from the environment to adjust the local tissue production of melatonin (Figure 2). Indeed, there are some pieces of evidence that suggest that pineal melatonin may, somehow, affect local extrapineal melatonin production.

In the rat retina, the melatonin content has been shown to increase after exogenous melatonin administration or after pinealectomy [57,58]. The former may indicate that the retina is able to uptake circulating melatonin, while the latter suggests that there is some type of control from the pineal gland over this extrapineal melatonin synthesis. Similar conclusions have been derived from the increase in melatonin content in the rat liver and brain after pinealectomy [111]. In the spleen, although the melatonergic biosynthetic pathway shows a greater expression during the diurnal phase, a melatonin peak occurs in the dark phase [70]. This suggests that, although enzymatic machinery for melatonin production is expressed in the spleen, its nocturnal melatonin content might be derived from the pineal gland. The thymus from adult rats, in contrast, shows a lower melatonin content at night, suggesting that high concentrations of circulating pineal melatonin may inhibit thymic melatonin synthesis. After pinealectomy, however, the thymic melatonin content increases [77]. All this evidence suggests that pineal and extrapineal melatonin maintain a complex relationship that is, in addition, tissue-dependent. However, despite the complexity of the possible mechanisms by which pineal melatonin may affect extrapineal melatonin production, we believe that a better understanding of this possible interplay will improve the outputs of the different approaches in the study of the actions of melatonin on different pathologies, including cancer.

## 4. Melatonin and the Cancer Process

The relationship between melatonin, as a chronobiotic hormone, and cancer has been studied for more than 40 years [112]. However, as illustrated in the next sections, melatonin presents a wide range of effects—from its appearance in unicellular organisms, where its antioxidant effect was predominant, towards the complexity of multicellular organisms, where several organs and organelles synthesize melatonin. In these organisms, melatonin functions include biological protection, ending in its function as a molecule that synchronizes the physiology with the night/day phase and its involvement in the promotion of sleep. With this big picture, it is easy to understand that melatonin functions in cancer may cover different angles and scales. In this section, we will take a tour around the different processes and stages of cancer where melatonin might be involved. As an important note, convey the idea that the roles to be described could be played by melatonin produced by the pineal gland or by local melatonin produced by extrapineal tissues.

The appearance of circadian rhythms in evolution was a way to anticipate cyclic changes to gear the body physiology to the external environment. Thus, the molecular clock and, therefore, cell function and survival, have been adjusted to dusk and dawn, seasonal and annual periods. The circadian system is mainly synchronized by the light/dark cycle, which, in natural conditions, is driven by the alternance of day and night. However, currently we usually spend daytime under low light intensities, while nights are excessively bright due to the use of artificial light at night (ALAN). Thus, human beings have the opportunity to extend or even shift our activity to the dark phase, thus invading the natural sleep time. This behavior sends an improper signal to our circadian system, which can result in a desynchronization of our biological processes that is known as chronodisruption (CD) [113]. CD manifests in several clinical symptoms, such as fatigue, mild to severe sleep disorders (insomnia), imbalanced appetite, and mood disorders (anxiety, depression, among others) [114,115]. Importantly, it has also been linked to a higher incidence of cardiovascular diseases, metabolic syndrome, premature aging, and cognitive impairment, and also with different types of cancer [116,117]. 

Indeed, epidemiological studies have revealed that CD increases the risk of developing several types of cancer. Shift work increases the incidence of breast cancer between 36–60% [118]. To cite some other examples, nasopharyngeal carcinoma and metastatic colorectal cancer have also been associated with CD [119]. In addition, it has been reported that the rate of prostate cancer can be higher in those males under persistent ALAN [120]. Additionally, a longer survival has been observed in patients with colorectal cancer whose behavior has a marked day-night circadian rhythm [121]. Patients with metastatic breast cancer have shown a poor prognosis when diurnal salivary cortisol do not show a ~24-h oscillation [122]. The increasing scientific evidence for the link between CD and cancer led the International Agency for Research on Cancer (IARC) to list shift work “as a probable human carcinogen type 2A” in 2007 [123,124].

**Figure 3:**
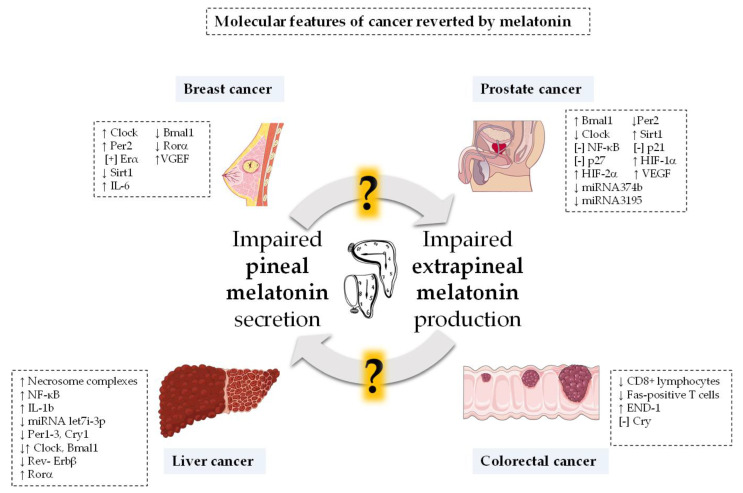
Schematic view of the possible dysregulation between pineal and extrapineal melatonin and the tumor molecular features reverted by the indolamine in breast, prostate, liver and colorectal cancer. The symbols stand for: ↑ upregulation or increment, ↓ downregulation, [+] activation, and [−] inhibition. This figure was built with SMART resources (Servier Medical Art), licensed under a Creative Common Attribution 3.0 Generic License. See http://smart.servier.com/. Prostate cancer image was extracted from Prostate Center Europe website: https://www.prostatecentereurope.com/disorders/prostate-cancer

There is compelling molecular evidence that both arrhythmic or suppressed melatonin secretion (i.e., low levels of melatonin at night) and CD correlate with the risk of developing several types of cancer, including those that are hormone-dependent (Figure 3). In this regard, it has been shown that serum melatonin levels below 39.5 pg/mL may increase the risk of breast cancer by 15 times compared to individuals with normal serum concentrations [125]. In support of this, another work has found that high levels of aMT6s in urine correlates with a lower risk of breast cancer incidence [126]. Consistently, a decreased concentration in aMT6s increases the risk of developing prostate cancer four-fold [127]. Finally, higher concentrations of melatonin in the blood are associated with a lower incidence of prostate and ovarian cancer [128,129]. 

In this regard, its chronobiotic function takes on paramount importance, since a desynchronized or suppressed melatonin secretion due to exposure to ALAN or to low levels of diurnal light can affect circadian synchronization in a great manner. However, as previously discussed in this review, it is essential to be aware of the importance of extrapineal melatonin, especially considering its direct antioxidant actions. Due to its relative recent appearance in the scientific knowledge and due to the technical difficulty in assessing its effects, there are not many studies that analyze the function of melatonin synthesized and secreted by other organs and tissues than the pineal gland. 

In the following sections we will review the functions of melatonin in key processes for tumor development, summarized in Table 1, as well as its reported actions in breast, prostate, liver, and colorectal cancer.

### 4.1. As an Antioxidant

Melatonin’s original function in unicellular organisms has been speculated to be antioxidant to prevent oxidative stress (see Section 2). In multicellular organisms, this role has been preserved, despite having acquired a wide variety of physiological actions, such as the control of circadian rhythms, sleep induction (in diurnal animals), or the regulation of seasonal reproduction and immune enhancement. In organisms, the balance between production and removal of free radicals is essential to keep health, so the mechanisms in charge of keeping them in moderate concentrations are important. Thus, the imbalance between free radical generation and removal leads to oxidative stress. This phenomenon results in macromolecular damage in DNA, among other macromolecules, which is involved in tumor development and cancerous growth [166]. Melatonin prevents injuries induced by oxidative stress at the molecular, cellular, tissue, organ, and organ system levels [167] through different actions, both directly as a free radical scavenger and indirectly by modulating antioxidant enzymes expression. Apart from these actions, melatonin also presents the ability to repair oxidized biomolecules [168].

Antioxidants can be classified according to the chemical routes through which they exert their actions against oxidative stress. Type I, also known as free radical scavengers, would be those antioxidants that directly react with free radicals and produce less reactive species that are harmless for biological targets, or end the radical chain reaction. Type II do not directly react with free radicals, but utilize different chemical routes. ^•^OH-inactivating ligands (OIL) are the most relevant Type II antioxidants. Type III, or fixers, are able to repair oxidatively damaged biomolecules mainly through H or electron transfer. Type IV are those that can exert their protection by a combination of the already mentioned effects, and also of other routes. According to this classification, melatonin has been suggested to belong to Type IV or multipurpose antioxidants. 

As a Type I, melatonin can detoxify several reactive oxygen species (ROS), such as hydrogen peroxide (H_2_O_2_), hydroxyl radicals (^•^OH), peroxyl radicals (ROO^•^), and singlet oxygen (^1^O_2_). Additionally, reactive nitrogen species (RNS) (e.g., nitric oxide radical (NO^•^) and peroxinitrite (ONOO^−^)), as well as hypochlorous acid [169] and a variety of free radicals (including ^•^OH, Br_2_^•−^, H^•^, ^•^OOCCl_3_, t-ButO^•^, G^•^, ^•^N_3_, ^•^NO, ^•^NO_2_, and SO_4_^•−^) can be detoxified by melatonin. Although the methoxy and amide side chains of the melatonin molecule also contribute to its antioxidant capacity, the reactive center of interaction with free radicals is located in the indole moiety, due to its high resonance stability and very low activation energy barrier towards the free radical reactions. An interesting phenomenon related to this antioxidant action of melatonin is the named ‘free radical scavenging cascade’, that starts after the interaction of melatonin with reactive species, generating intermediates that are, in turn, free radical scavengers with different efficiencies and specificities (reviewed in [170]). Thanks to this cascade, melatonin can scavenge up to four or more reactive species, becoming a very effective antioxidant, even several times more effective than vitamin C [171] or E [172] at equivalent dosages. Some of these metabolites have been extensively reviewed in terms of their antioxidant activity by Galano and Reiter (2018) [170], and they are: N-acetylserotonin (NAS), 5-methoxytryptamine (5-MT), cyclic 3-hydroxymelatonin (c-3OHM), N1-acetyl-N2-formyl-5-methoxykynuramine (AFMK), N^1^-acetyl-5-methoxykynuramine (AMK), *6-hydroxymelatonin* (6OHM), 4-hydroxymelatonin (4OHM), 2-hydroxymelatonin (2OHM). 

As a Type II, melatonin is able to quench singlet oxygen (^1^O_2_) [173,174], also chelating different metal ions [175] and decreasing the amounts of free radicals produced by the interaction of Cu(II), Fe(II), Zn(II), Al(III), and Mn(II) with the β-amyloid peptide [176]. Melatonin also inhibits Cu-mediated lipid peroxidation [177], and Cu(II)/H_2_O_2_^−^ induced damage to proteins [178]. It has been suggested that melatonin may also prevent the Cu-induced generation of free radicals in vivo by binding this metal [179].

As a Type III antioxidant, melatonin can regenerate glutathione, ascorbic acid, and Trolox through an electron transfer processes, improving their antioxidant effects [180,181]. Melatonin is also capable of repairing biological molecules such as oxidized DNA [29,182,183], which has been explained considering its ability to transform guanosine radical to guanosine by electron transfer [168].

Apart from these antioxidant actions as Type I, II, and III, melatonin also exerts its antioxidant effects through the enhancement of the DNA repair machinery [184,185,186,187], the activation of antioxidant enzymes (mediated by calmodulin, which downregulates the activity of the RORα melatonin receptor, influencing the expression of NF-κB-induced antioxidant enzymes [188,189,190,191,192,193,194]) or the inhibition of pro-oxidative enzymes [195,196] in normal cells. This antioxidant activity has been related to MT3-mediated response (membrane receptor-independent) [197]. However, and interestingly, it has been demonstrated that in cancer cells melatonin enhances free radical generation, becoming pro-oxidative [198,199]. Therefore, and as previously stated, the actions of melatonin are context-specific.

Therefore, these unique antioxidant features (e.g., free radical scavenging cascade and context specificity) make melatonin a central molecule from the antioxidant perspective, especially related to cancer prevention and treatment. In this sense, it is important to mention that extrapineal melatonin acquires an important role in terms of antioxidant and anti-inflammatory properties, since these processes require higher concentrations than those reached by the pineal melatonin. Thus, the redox status of the cell is likely to exert additional control on extrapineal melatonin synthesis, although pineal melatonin may contribute, somehow, to this regulation [5]. We insist, therefore, on the importance of unravelling the possible interplay between pineal and extrapineal melatonin regulation.

#### Antioxidant Actions and Genome Stability

Cancer cell biology is under redox control at any of its steps, such as proliferation, migration, invasion, vascularization, and metastasis [200]. In addition, the tumor microenvironment is enriched in superoxide, hydrogen peroxide, and nitric oxide molecules produced by cancer cells, extracellular matrix, immune cells, and by external insults such as UV light or ionizing radiation. Thus, targeting redox status has been a main field to study in cancer research. As aforementioned, melatonin detoxifies oxidant molecules by the indole moiety, that is considered the reactive center of interaction with free radicals, or by modulating the activity of antioxidant enzymes.

The production of H_2_O_2_ by cancer cells is mainly due to the metabolic switch from OXPHOS to glycolysis, known as Warburg effect. This shift towards a glycolytic status depends on NO production by dendritic cells when they are active in the extracellular matrix [201]. H_2_O_2_ is also produced by apoptotic cancer cells due to the anaerobic conditions and the lack of nutrients. The hydroxy peroxide stimulates growth factor receptors (GFR) (epidermal GFR, insulin-like GFR, transforming GFR beta, platelet-derived GFR, etc.), driving the activation of Ras-Raf-Erk and the PI3K-Akt pathways and inducing proliferation [200]. Interestingly, PI3K-Akt increases the expression of glycolytic genes (*Glut1*, *Hk2*, *Pfkfb3*, and *Ldha*) and PDK, which suppresses the Tricarboxylic Acid cycle, which in turn elevates H_2_O_2_ production [202,203,204]. The nicotinamide adenine dinucleotide phosphate (NADPH) oxidases (NOX) and dual oxidases (DUOX), that produce O_2_**^.^**^−^ and H_2_O_2_, have been found as a major source of oxidants in cancer cells. NOXs enzymes are also present in such as colon cancer cells (where they regulate proliferation) or EBV-infected gastric cancer cells (where they regulate cell progression). DUOXs enzymes seem to regulate epithelial mesenchymal transition (EMT), invasiveness, and the induction of endothelial growth factor (VEGF) and hypoxia-inducible factor 1-alpha (HIF-1α) in pancreatic adenocarcinoma cell line. Interestingly, a direct effect of melatonin in PI3K-AKT-mTOR pathway is demonstrated when it is combined with Endoplasmic Reticulum-stress (ER-stress) agents (thapsigargin or tunicamycin), inhibiting melanoma cell lines growth [205]. 

Several studies have demonstrated that melatonin maintains genome stability by scavenging ROS or activating the DNA damage repair system. Melatonin (50 mM) and its metabolite N1-acetyl-N2-formyl-5-methoxykynuramine administration reduce DNA damage (DD) under H_2_O_2_ treatment. In extracts of human skin, melatonin protects against UV irradiation-induced DD by activating antioxidant enzymes (SOD, GPx, CAT). Moreover, melatonin is able to potentiate different pathways of DNA repair, including base excision, mismatch and nucleotide excision repair, homologous recombination, and nonhomologous end-joining [130]. Another study on genome-wide expression microarray analysis treats MCF-7 cells with melatonin and methyl methanesulfonate (a DNA damaging agent) and demonstrates an upregulation of DD repair (DDR) genes when compared to the untreated control group [185]. Other indirect functions of melatonin in DDR, widely reviewed in [131], have been described such as enhancing mitochondrial activity, pro-oxidative enzyme inhibition, and glutathione synthesis activation, among others. 

### 4.2. As a Chronobiotic

Melatonin is considered a chronobiotic, which could be defined as “a substance that adjusts the timing of the central biological clock”. Physiologically, melatonin secreted by the pineal gland is an important output and signal of the circadian system. Its secretion is rhythmically controlled by the SCN, entrained by the light–dark cycle, and it is mainly produced at night (for details, see Section 3.1.1). Apart from this circadian regulation, pineal melatonin secretion can be acutely suppressed by light at night, especially at 460–480 nm. To understand how melatonin exerts its chronobiotic action, circadian system function will be reviewed along this section:

#### 4.2.1. Circadian Clock Functioning

**Figure 4:**
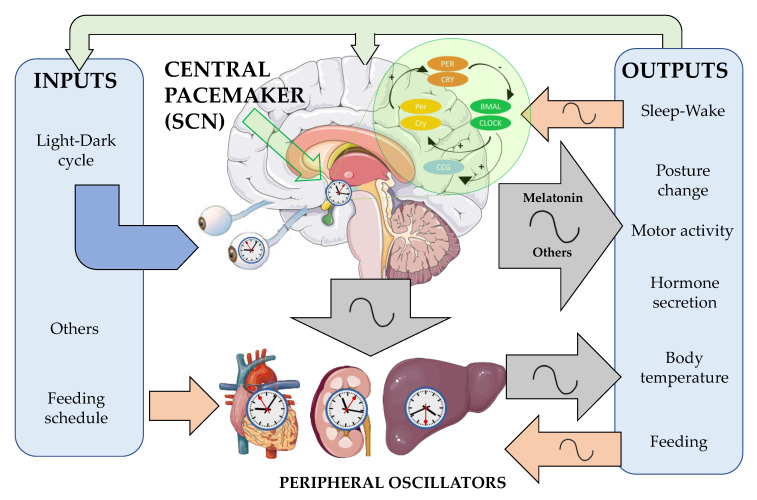
Schematic organization of the circadian system, with the central pacemaker located in the suprachiasmatic nuclei (SCN) and peripheral oscillators in most organs and tissues. The system presents inputs, with the light–dark cycle being the most important synchronizer. Sleep–wake cycle, hormone secretion, and body temperature are just some of the outputs of the circadian system. These outputs can act on the clock itself or modify the inputs in a feed-back manner. The molecular clock machinery is also represented in the greenish circle, with the positive (BMAL and CLOCK) and negative (PER and CRY) elements, also affecting the expression of clock-controlled genes (CCG). Modified from Garaulet and Madrid (2009) [219]. This figure was built with SMART resources (Servier Medical Art), licensed under a Creative Common Attribution 3.0 Generic License. See http://smart.servier.com/.

Mammals’ circadian system consists of a hierarchically organized structure of oscillators distributed over most organs and tissues (Figure 4). The suprachiasmatic nuclei (SCN) acts as the central pacemaker, while the peripheral clocks oscillate under its control. Their rhythms are generated by a transcriptional-translational feedback loop between positive and negative groups of clock genes (CG). As positive elements, circadian locomotor output cycles kaput (*Clock*) and brain and muscle aryl hydrocarbon receptor nuclear translocator-like (*Bmal1*) promote the synthesis of two transcription factors which, after heterodimerization, induce the expression of negative components of the molecular circadian clock: Period (*Per 1*, *2*, *3*), Cryptochrome (*Cry1* and *Cry2*) and a nuclear receptor subfamily 1 (*Rev-Erbα*) [206,207]. *Chrono*, a recently described clock gene, seems to act as a transcriptional repressor of the negative feedback elements in the mammalian clock [208]. Additionally, three retinoid-related orphan receptor (Rorα, β and γ), two reverse- erythroblastosis (Rev-Erb α and β) and two casein kinases 1 (CK1δ and ε) are assembled in this complex regulatory system [209]. In addition, this core oscillator system is associated with several often tissue-specific accessory proteins that also undergo circadian regulation. Among them, nicotinamide phosphoribosyltransferase (NAMPT) [210], peroxisome proliferator-activated receptor-γ (PPARγ) [211,212], sirtuin 1 (SIRT1) [213,214], AMP-activated protein kinase (AMPK) [215], and protein kinase Cα (PKCα) [216,217] are of particular relevance, connecting oscillators with metabolic sensing and mitochondrial function. All of them are controlled or modulated by melatonin [218], which confirms that this molecule as a key factor in the connection between circadian oscillators and health maintenance, which extends to the prevention and suppression of cancer. 

#### 4.2.2. Importance of the Light-Dark Cycle and Melatonin Rhythm

This system of oscillators requires different inputs to be reset every day (Figure 4), since otherwise it tends to delay (endogenous period > 24 h). The most important input for the central pacemaker is the light–dark cycle, which is also closely related to the regulation of pineal melatonin secretion. Other inputs are social contacts, physical exercise, and meals, especially relevant for peripheral oscillators. This structure also displays outputs that are measurable, with rhythmic melatonin secretion by the pineal gland being of relevance, also involved in other outputs such as sleep–wake cycle in diurnal animals and thus motor activity, body posture, and other hormone secretion rhythms, among others. Interestingly, some of these outputs can also act as inputs on the system, with melatonin being a good example of these feedback regulation processes [220].

Indeed, melatonin is importantly involved in the SCN-melatonin feedback loop. Thus, melatonin secreted by the pineal gland during the dark phase is transported through cerebrospinal fluid, reaching the SCN, which expresses membrane melatonin receptors (MT1 and MT2). It has been shown that melatonin phase-shifts *Bmal1* and *Rev-erbα* expression [221], probably through these receptors expressed in the SCN [222]. Additionally, independently of MT1 and MT2 receptors [223,224,225], melatonin exerts an inhibitory effect on glutamatergic activity in several areas of the brain, therefore modulating the glutamatergic activation of the SCN conveyed by the retinohypothalamic tract. Thus, melatonin exerts a double mechanism of action: (i) an acute inhibitory effect on neuronal firing mediated by glutamatergic and or glutamatergic-related events, and (ii) the phase shifting of the clock. This double regulation exerted by melatonin in regulating the functioning of the main pacemaker points out to considering this molecule as a central signal within the circadian system, with relevant functions.

Thus, nocturnal secretion of melatonin acts as an emissary that distribute, via the general circulation, the nocturnal/circadian message through the entire body. Although the redundancy within the circadian system is elevated and the circadian signal can be transmitted by different nervous or hormonal clock outputs, there are structures that only rely on the melatonin signal to temporally organize certain responses [226]. Thus, a synchronized melatonin secretion is of special relevance in terms of health maintenance. However, the aforementioned redundancy makes difficult to demonstrate in vivo the possible effect of melatonin on the autonomous circadian expression of genes or proteins. In this sense, there is certain evidence that melatonin could be responsible for circadian activity in several cells or tissues, such as rhythmic protein synthesis in hepatocytes in vivo [227] or in primary isolated adipocytes [228], as well as in the circadian modulation of sodium-potassium-ATPase and sodium-proton exchanger in human erythrocytes [229]. 

#### 4.2.3. Melatonin, Clock Genes and Cancer

Considering the close relationship between clock genes, cell cycle regulation, and survival and repair mechanisms [230], the importance of melatonin in cancer evolution is not surprising, not only in terms of its antioxidant effects, but also with regard to its chronobiological implications. 

Several studies carried out in tumors of different nature, including breast, prostate, colon, lung or liver cancer, among others, show that clock genes promoters are usually methylated (although other types of mutations have been found in tumors), and therefore silenced [231,232] along the tumor process. On the contrary, it is interesting to highlight that the overexpression of *Per2* is able to abolish cell proliferation both in vitro and in vivo [233,234], probably by controlling cell cycle genes (*Myc*, *Cyclin d1*, and *Wee1*) [235]. Regarding Bmal and Clock, opposite roles for them have been described, with several evidences demonstrating that *Bmal1* acts as a tumor suppressor, while *Clock* would be a tumor driver. This duality in the role that clock genes exhibit supports that their functions are tissue-dependent. 

The way in which melatonin regulates clock genes expression has been demonstrated through different approaches. Melatonin signaling, in an MT2-receptor-dependent manner, is implicated in the post-transcriptional regulation of PER1 and CRY1 at the SCN [134,135]. Other studies performed in Pars tuberalis have reflected that melatonin exerts a transcriptional control of *Per1* and *Cry1* genes in a time-dependent manner and that MT1 deletion causes reduced expression of *Cry*, *Bmal1*, and *Clock* genes. Moreover, studies carried out in the mouse retina demonstrate that in the absence of MT1 receptor, mRNA of Per1, Cry1, and Bmal1 decreases just at the beginning of the dark phase [136]. Interestingly, in pinealectomized chicks the hepatic expression of clock genes were decreased, highlighting the importance of pineal melatonin in regulating peripheral clock genes such as the one located in the liver [236]. Other studies have pointed out that melatonin can act via nuclear orphan receptor genes as a second messenger to regulate core clock genes. 

Recent advances indicate that melatonin can interfere, in a variety of tissues including the SCN [237], with the ubiquitin-proteasome system required for the clock proteins’ “precision time”. As reviewed by Vriend and Reiter, melatonin presents many similarities with bortezomib, a proteasome inhibitor. Therefore, melatonin could provide selective stability to several proteins, especially BMAL1, whose expression increases during the dark phase as melatonin does [137]. Therefore, high levels of melatonin at night would increase the availability of BMAL1 through reducing its proteasomal cleavage, eventually enhancing the levels of CRY, PER, and REV-ERBα [137,138]. 

For a long time, melatonin has been related to SIRT1, the class III chromatin remodeller, by histone deacetylation. Considering that SIRT1 seems to be an antagonist of the deacetyl transferase CLOCK that deacetylases BMAL1, it is expectable that it also modulates the activity of CLOCK/BMAL1 heterodimer [238]. In addition, CLOCK/BMAL1 regulates the expression of *Sirt1* through the E-box element, closing the regulation loop [239]. On the other hand, tumors that express CLOCK and BMAL1 show an upregulation of *Sirt1* that induces the deacetylation of transcription factors leading to cell division. It has also been found that melatonin decreases the expression of *Bmal1*, *Clock* and *Sirt1* in cancer cells, with an antiproliferative function [139]. Although the exact molecular mechanism by which melatonin regulates SIRT1 is not known, considering the role of SIRT1 in the protection from ROS (through scavenging functions), it has been suggested that melatonin could downregulate *Sirt1* by keeping low levels of ROS [132]. However, a direct effect on this regulation cannot be discarded [133].

The aforementioned data demonstrate the direct regulation of clock genes expression by melatonin. This may indicate that an impairment in melatonin secretion could provide the necessary conditions for tumor growth.

### 4.3. As an Immunomodulator and Anti-Inflammatory

Melatonin is considered as an immunomodulatory factor, although a complete understanding on the mechanisms by which melatonin regulates immunity has not been achieved yet. Most data suggest that melatonin would act as an immune buffer, exerting stimulant actions under basal or immunosuppressive conditions or acting as an anti-inflammatory component under exacerbated immune responses, such as acute inflammation. The relationship between the neuroendocrine and immune system is well documented and exemplified by the bidirectional communication between the products of both systems. Within this network, we also find melatonin and the pineal gland, whose link with immune system has been widely explored through mainly (i) pinealectomy, which produces weight loss in lymphoid organs and a decrease in innate and specific responses; and (ii) association between circadian and seasonal adjustment of immune system and melatonin synthesis [65,240]. 

This bidirectional link is also illustrated by the fact that the pineal gland is a target of the immune system, with many immune components and products interacting with melatonin. Indeed, some cytokines, interferon-gamma (IFN-γ) [241], granulocyte-macrophage colony-stimulating factor (GM-CSF), and granulocyte colony-stimulating factor (G-CSF) [242], are able to stimulate melatonin secretion, while IL-1 [243] may inhibit it. Other organs or factors that have been related to melatonin system are the bursa of Fabricius [244] or TNF-α (both directly or indirectly through lipopolysaccharide (LPS)), all these mechanisms reviewed in [72]. For its part, melatonin can stimulate the release of IL-2 via up-regulation of MT1, eventually leading to an increase in natural killer (NK) cells [72]. Melatonin also enhances antigen presentation by macrophages to T-lymphocytes, which leads to the activation and proliferation of cytotoxic T lymphocytes. This action is also favoured by melatonin through triggering the release of cytokines such as IFN-γ, TNF-α and IL-6 as well as suppression of IL-4 [140], whose role at the first stages of tumor development is protective. Recently, melatonin has been shown to suppress eosinophils and Th17 cells in hamsters infected and treated with a chemical carcinogen [141]. In addition, as previously described in this review, melatonin is produced by cells and organs of the immune system, which also present melatonin receptors. Although knowledge of the physiological actions of local immune-derived melatonin is limited, there are some pieces of evidence in this regard. Carrillo-Vico et al. firstly described the modulation of IL-2/IL-2 receptor system exerted by local melatonin via receptor-mediated intra-, auto- and/or paracrine actions [67]. Additionally, low levels of Hydroxyindole-O-methyltransferase (HIOMT, a synonym for ASMT) activity and MT1 expression have been found to decrease IL-2 response [245]. In addition, local melatonin has been shown to be involved in the modulation of the phagocytic capacity of the colostrum immunocompetent cells [246], in accordance with the high melatonin concentrations found in stimulated peripheral blood mononuclear cells [66]. Those high concentrations also impair exogenous melatonin action in the production of IL-2, probably by saturating binding sites [67]. 

As previously underlined, knowing the possible interplay between pineal secretion and local melatonin production/levels is of great interest in terms of the effects of this molecule on the immune system. In this sense, the functions of NF-κB in the transcriptional control of *Aanat* expression have been found to be opposite in pinealocytes vs. macrophages, which may represent a switch mechanism in the regulation of pineal versus immune-derived melatonin under inflammatory conditions [247]. 

#### Melatonin and Immune Evasion in Cancer

The ability of cancer cells to escape from the immune system is one of the advantages that cells acquire during the transformation process. As aforementioned, melatonin has anti-inflammatory and immunomodulatory effects, these two properties being intimately linked with cancer biology. 

The innate immune system is the first barrier that tumor cells find against its survival. Although monocytes are also known to play an important role in adaptative immunity, they have a central role in activating innate immunity by inducing inflammation under external stimuli. In this regard, melatonin treatment has been demonstrated to present an effect in monocytes and macrophages, driving the secretion of pro-inflammatory cytokines [248,249]. NK cells are a type of cytotoxic lymphocytes that are critical to the innate immune system and have a well described antitumor effect. Melatonin increases the total number of NK cells and monocytes by enhancing the antitumor effect of IL-2 in melanoma cell lines.

Regulatory T cells (Tregs, that are CD4(+) CD25(+) FoxP3(+)) are part of adaptative immunity and have a role in maintaining self-tolerance. Tregs are decisive, not only in the protection against destruction of own tissues by autoimmune immunocompetent cells, but also in the immunological response to tumor cells. Tregs could be responsible for the progression of acute and chronic leukemias [250] and have been found increased in patients and animal models of gastric cancer. In this regard, in vivo melatonin administration mediates the downregulation of Foxp3 in Tregs, thus decreasing Treg infiltration in tumor areas as well as reducing tumor growth. In addition, CD4(+) T helper cells are also activated by melatonin through the increased production of IL-2, IL-6, IL-12, and interferon gamma (IFN-γ) via nuclear melatonin receptor RZR/ROR [142]. 

### 4.4. Melatonin and Proliferation

Proliferation is a process tightly regulated by well-known checkpoints that can be triggered by several pathways in the cell. Indeed, the ability to sustain proliferation is a hallmark of cancer cells. In this regard, the effect of melatonin has been shown in several signaling pathways involved in proliferation, including cyclin-dependent kinases (CDKs), PI3K/AKT, estrogen receptor (ER) signaling (see Section 5.1) and telomerase.

CDKs are a family of protein kinases that regulate the cell cycle. Several studies demonstrate that melatonin blocks the progression through G1-S by inhibiting the transcription of cyclins and CDKs, including *Cyclin d1*, *Cyclin b1*, *Cdk4*, and *Cdk1* [143,144,145,146], in osteosarcoma and breast cancer cell lines. 

In breast cancer cell lines, melatonin strongly inhibits the phosphorylation of PI3K, AKT, PRAS40, and GSK-3 proteins, driving an inactivation of the PI3K/AKT signaling pathway. Conversely, PI3K, AKT inhibitors or akt-specific siRNA block melatonin-induced inhibition of proliferation [147]. In support of that, vitamin D3 and melatonin, when simultaneously administered to MCF7 breast cancer cells, can induce TGFβ activation, a reduction in AKT phosphorylation and MDM2, increasing the p53/MDM2 ratio and inducing cell cycle arrest, suggesting a synergic effect [148]. 

Telomerase avoids telomere shortening, keeping their length under division. This enzyme is inactive after birth, except in stem cells or in cancer cells, which permits them to sustain proliferation [149]. Interestingly, melatonin inhibits telomerase activity in a dose-dependent manner [150], probably by inhibiting Tert, the catalytic subunit of the enzyme [251]. The lack of Tert in tumor cells drives telomerase inactivation and therefore telomeres attrition, leading to cell arrest.

### 4.5. Melatonin and Apoptosis

The property of acquiring resistance to apoptosis by cancer cells is considered one of the important hallmarks of cancer [252]. Melatonin has been reported in several studies to have the ability to block this capacity through several mechanisms. According to different studies, melatonin treatment can decrease BCL2 and increase BAX levels in pancreatic carcinoma and human myeloid leukemia cells lines [151,152]. This ratio is an important marker, since BCL2 has a relevant role in conferring resistance to apoptosis, while *Bax* is a proapoptotic gene. In addition, other studies have reported the melatonin-mediated upregulation of p53, p21, and cleavage-caspase [153]. Additionally, as widely reviewed in [253], melatonin is able to inhibit NF-κB upstream genes (*Myd88* and *Trif*). This pathway is usually upregulated in cancer cells and induces apoptosis resistance, probably via inhibiting the TRAIL pathway, TNFR, and FASL, or activating apoptosis inhibitors (cIAP-2, XIAP, and survivin). Another suggested mechanism for melatonin to avoid tumor cells apoptosis resistance points to its role in regulating homeostasis between apoptosis and autophagy through the enhancement of mammalian sterile 20-like kinase 1 (MST1), a protein that reduces ROS content in the cell [154].

Additionally, as mentioned in other sections, melatonin acts via the PI3K/AKT/mTOR pathway, triggering the activation of RAS/MEK/ERK. Interestingly, in normal cells melatonin activates AKT, while in cancer cells this interaction turns to an inactivation due to a switch in the MT coupling of G-protein [254,255]. This alternative G-protein coupling leads to an inhibition of the MAPK family protein, triggering apoptosis, as has been shown in gastric cancer cells where melatonin activates pro-caspase enzymes through the activation of p38 and JNK as well as through NF-κB suppression. Due to the tissue-dependent role of the MAPK family proteins, the relation of melatonin to MAPK not only depends on the tumor status of the cell but also on the type of cell where this is occurring [155,156].

### 4.6. Melatonin and Angiogenesis

The novo formation of a vasculature network is an event required in cancer cells to keep them supplied with oxygen and nutrients. Several vascular factors are involved in this complex process, including endothelial growth factor (VEGF), epidermal growth factor (EGF), hepatocyte growth factor (HGF), and platelet-derived growth factor (PDGF). The roles of melatonin in both the inhibition of VEGF (or the induction of VEGF in low levels in blood) and the disruption of cancer neo-angiogenesis have been reported in several studies that have been previously reviewed [256]. 

Among other cell functions, including erythropoiesis and metabolism, HIF-1α induces neo-angiogenesis in tumors. When oxygen levels are low, HIF-1α translocates into the nucleus and heterodimerizes with ARNT, inducing the expression of angiogenic genes. Several studies have indeed proven the effect of melatonin inhibiting HIF1α. First of all, melatonin is able to inhibit the AKT/glycogen synthase kinase-3β (GSK-3β) signaling pathway, which is required for HIFα stabilization [157]. Secondly, a direct effect of melatonin in HIF1α has been described under hypoxia [158]. However, considering the complexity and tissue-dependence of melatonin functions, it is difficult to generalize these specific roles. Therefore, it is not surprising that there are studies that confirm the role of melatonin in HIF-1α and VEGF, while others in different cancer cell lines have not been able to confirm this relationship.

### 4.7. Melatonin and Metastasis

The metastatic form of a tumor represents the most aggressive step of cancer biology. Metastasis drives several events, including angiogenesis, loss of cell–cell contacts, extracellular matrix remodeling, anoikis evasion, tissue invasion, intravasation, transport around the body, and the extravasation/establishment of a secondary tumor.

Several studies have demonstrated that the loss of cell–cell contact by a downregulation of anchoring proteins can be blocked by melatonin. In this sense, it has been demonstrated that melatonin upregulates (i) E-cadherins in breast cancer and metastatic cancer [159,160], (ii) occludins in the A549 lung adenocarcinoma cell line [161], and (iii) integrins in glioma and breast cancer cells [162].

In addition, melatonin is also able to reduce the expression/activity of metalo-proteases (including MMP-9 and MMP-2) in the extracellular matrix remodeling of a myriad of cancer cell lines (gastric, breast, renal, and oral cancer and nasopharyngeal carcinoma) [163]. Moreover, this indoleamine can rearrange the cytoskeleton, probably via vimentin downregulation [164]. 

Finally, melatonin has been also shown to inhibit EMT in gastric cancer cell lines by interfering with NF-ĸB, downregulating *Snail* and *Slug*, and attenuating the Wnt/β-Catenin pathway [165]. 

## 5. Melatonin: Reported Actions in Breast, Prostate, Liver, and Colorectal Cancer

### 5.1. Breast Cancer

According to GLOBOCAN [257], the estimated number of breast cancer new cases worldwide reached more than 2 million women, representing 1 out of 4 cancers diagnosed in females. Past studies concluded that the existence of a high incidence of breast cancer in urban areas in north vs. south latitudes could be due to a deficit in vitamin D [258]. In 1896, Beatson re-focused this issue by demonstrating a tumor reduction after bilateral ovariectomy in a premenopausal patient with advanced breast cancer. Later, several studies led to establishing a possible relationship between melatonin and hormone-dependent breast cancer (HDBC) considering the following findings: (i) countries with a lower incidence of pineal gland calcification show lower ratios of HDBC, (ii) melatonin receptors have been identified in ovaries, and (iii) patients with HDBC present lower plasma melatonin amplitude than those with non-hormone-dependent breast tumors and healthy controls [259]. These observations have been supported by the fact that a lower incidence of breast cancer has been described in blind when compared to sighted women exposed to ALAN, which suggests a putative “protective effect” of melatonin in this type of cancer [258] (higher plasma concentrations in blind people due to the lack of direct light suppression). This hypothesis has been supported by several epidemiological studies. Among this evidence, a positive correlation between the total time spent on shift work and the incidence of breast cancer has been established [260]. These results could indicate that the decrease in plasma melatonin levels may drive an increase in reproductive hormones such as estradiol, eventually driving HDBC, as discussed below. 

In vivo experiments in pinealectomized rats exposed to short photoperiods and treated with the carcinogen DMBA show a reduction in HDBC compared to non-pinealectomized control rats. Accordingly, rodents treated with DMBA and melatonin produce a reduction in the expression of estrogen receptor-α (ERα) at the tumor level [261]. Therefore, the protective effect of melatonin in HDBC could be due to the regulation that melatonin may exert on estrogen receptors or by controlling their signaling pathway. On the other hand, it is known that melatonin is a calmodulin antagonist that competes for the binding site in ER. Thus, melatonin may interfere the estradiol-transcriptional activation of estradiol-responsive genes, inhibiting its binding to DNA at two types of promoter elements: estrogen response (ERE-) and activator protein 1 (AP1-) elements [262]. 

The action of melatonin on clock gene expression has been extensively documented by de Almeida Chuffa [263] and is summarized in Table 2. Breast cancer cells have dropped the expression of *Bmal1*, via MT1-RORa1, which is described as acting as a tumor suppressor by inducing the downregulation of *Sirt1* [264]. Melatonin can also act on RORα receptor, inhibiting the 5-lipoxygenase gene expression and blocking the proliferation of MCF-7 breast cancer cells. However, the *Clock* gene is usually activated in HDBC, inducing a pro-proliferative activity mediated by E2-ERa signaling [265]. Other biological processes, including metastasis or metabolic switch, have been also shown to be regulated by melatonin in breast cancer cells. Melatonin is able to inhibit IL-6 and VEGF secretion by blocking the stimulation and matrix reorganization, a necessary step for the metastatic process [266,267]. Finally, as a consequence of metabolic changes and uncontrolled proliferation, mitochondrial disfunction is a feature of cancer cells. In this sense, MCF-7 breast cancer cells treated with melatonin exhibit a decrease in ATP production and an upregulation of complex III activity when treated with melatonin catabolite *6-hydroxymelatonin* [268,269].

### 5.2. Prostate Cancer

Another common hormone-dependent tumor is prostate cancer (PC). This is one of the leading causes of cancer death worldwide in males. Several epidemiological studies have demonstrated that shift workers present a higher risk of developing PC (by approximately 24%). In addition, impaired sleep duration or sleep quality as well as sleep disorders such as insomnia may also increase the risk of developing PC [284]. Interestingly, men with non-metastasized prostatic carcinoma show arrhythmic melatonin secretion. In addition, plasma melatonin concentration has been found to present an inverse correlation with PC prevalence, and the intake of oral exogenous melatonin is also related to a better prognosis of the disease. Melatonin has shown diverse effects in PC, such as proapoptotic, antiproliferative, antioxidant, anti-inflammatory/immunostimulatory, and antiangiogenic actions, all of which are widely reviewed in [285]. 

The fact that melatonin presents a potent antigonadotropic effect could shed light on the understanding of melatonin actions in PC. In order to gain knowledge of this process, several studies have been conducted, indicating that the administration of melatonin at different concentrations can suppress tumor growth [286] by: (i) inducing cell cycle arrest in the G0–G1 phase [287,288] or (ii) the activation of intracellular PKA and PKC followed by an inhibition of NF-κB and the transcriptional activation of p21 and p27 [289], partially in an MT1-dependent manner [290,291]. 

Although the aforementioned are the most studied mechanisms, other pathways by which melatonin may exert a protective effect in prostate cancer have been described. In this sense, melatonin inhibits *Sirt1* in prostate cell lines usually upregulated in prostate cancer [139]. Other authors conclude that the effect of melatonin in PC is exerted by limiting glucose uptake in these tumor cells [292]. Additionally, a potent anti-angiogenic effect exerted by melatonin has been described under hypoxia and is mainly mediated by decreased levels of Hif-1a, Hif2a, and Vegf mRNA and the upregulation of several miRNAs (miRNA374b and miRNA3195) [293]. Last, but not least, the indolamine can also act as a chronobiotic agent in PC cells, resynchronizing clock genes’ expression through *Per2* and *Clock* upregulation and *Bmal1* downregulation [275] (Table 2). 

### 5.3. Liver Cancer

Liver physiology and pathophysiology are subjected to a tight circadian regulation, mostly influenced by feeding time [294]. Circadian disturbances, such as alterations in the sleep–wake cycle and disrupted patterns of hormones secretion are common risk factors associated with liver cirrhosis, a previous step to develop liver cancer (LC) [295]. Hepatocellular carcinoma is the most abundant type of LC and the fourth leading cause of cancer death worldwide according to the World Health Organization (WHO) [296]. Additionally, some estimations predict that LC will increase by around 62% from 2018 to 2040. The estimated number of deaths caused by this type of cancer is also expected to increase more than 64% within the same period [297].

Although less studied than the previously reviewed cancers, there is some evidence that chronodisruption, melatonin, and liver cancer are interconnected. Interestingly, a role for melatonin has been demonstrated in liver injuries, as reviewed in [298]. Considering the wide range of melatonin functions, it is not surprising that this molecule can exert different effects in liver cancer, acting as an antioxidant and anti-inflammatory molecule, reducing apoptosis rate, inhibiting necrosis, suppressing autophagic cell death, preventing steatosis, reducing neutrophil infiltration, improving the hepatic detoxification system, and attenuating mitochondrial damage, among others [298]. Similarly, melatonin effects have been also demonstrated in previous stages of liver disease before cancer development. As an example, melatonin protects against high fat diet (HFD)-induced hepatic steatosis in mice and rats [299] and is also able to reduce non-alcoholic-fatty liver (a disease induced by HFD) in rats, probably by protecting against oxidative stress and inflammation [300]. In addition, melatonin also protects the liver from developing fibrosis and cirrhosis, probably by attenuating (i) the formation of the necrosome complex and (ii) NF-ĸB and pro-inflammatory cytokines, including TNF-α and IL-1β, by Kupffer cells [301,302,303]. 

The protective effect of melatonin on the development of HCC is demonstrated by the fact that it protects from the stages prior to liver tumor development (liver injuries, steatosis, NAFLD, fibrosis, cirrhosis, as mentioned previously). Studies with human biopsies of patients with HCC confirm this effect with the finding of a correlation between Single Nucleotide Polymorphisms (SNPs) in MT1 and MT2 and a higher risk of developing HCC [304]. Among other evidence supporting this protective effect of the indoleamine on the development of HCC is the fact that melatonin can inhibit the proliferation, migration, and invasion capacities of Huh7 and HepG2 hepatoma cell lines by inducing the expression of the miRNA let7i-3p, which reduces *Raf1* expression, eventually reducing the activation of mitogen-activated protein kinase signaling downstream from RAF1 [305]. On the other hand, it is well known that diethylnitrosamine (DEN) treatment induces liver cancer, in part by deregulating clock genes through an increase and decrease in the expression of *Clock*-*Bmal1* and *Per1-3*-*Cry1*, respectively. Interestingly, melatonin treatment is able to revert this DEN-induced clock gene deregulation [279] (Table 2).

### 5.4. Colorectal Cancer

Colorectal cancer (CRC) represents the third most deadly and fourth most commonly diagnosed cancer in the world according to the WHO [306]. Although CRC development is linked to a genetic profile, including WNT and MYC activation and *Kras* and *Apc* mutations, among others [307], this type of cancer is also closely associated with the individuals’ lifestyle, including diet, sleep, and physical activity. Thus, in developed countries, where obesity, sedentary lifestyle, red meat consumption, alcohol intake, smoking habit, and ALAN are usually present, the incidence is rising [308]. In terms of chronodisruption, different studies have demonstrated that patients with CRC present lower levels of plasma melatonin than healthy individuals [309]. In addition, nurses who have worked 3 days/week for more than 15 years show an increased risk of developing CRC, evidencing the importance of the chronobiotic function of melatonin in intestine functioning, probably due to its synchronizing effect on this peripheral clock. In this regard, the colon epithelium is composed of highly proliferative cells whose doubling time follows a circadian pattern [310]. An example of the importance of the coordination between cell cycle and circadian rhythms is the protection against DNA damage. At a certain point of the cell division, the DNA is more susceptible to being damaged, since its structure is more unfolded due to its euchromatic conformation. If the temporal organization of the cell cycle is uncoordinated with the circadian rhythm, the DNA can be damaged by agents derived from metabolism and other biological processes, and even the DDR program may not be ready (transcribed, translated, or DDR protein-activated) for repairing that damage. Thus, keeping the clocks geared helps to protect the cell from damage. The importance of circadian rhythmicity goes beyond the mentioned mechanisms since, in addition to being a risk factor for CRC, *Clock*, *Per*, and *Bmal1* have been found to be modified in patients with colorectal cancers [280,281,282,283] (Table 2). 

Regarding melatonin and CRC, several in vivo studies have demonstrated its anti-tumor effect. As an example of this evidence, rats treated with melatonin at 20 mg/L (oral administration) [311] or 1 μg/animal (subcutaneous) [312,313] develop less CRC under 1,2-dimethylhydrazine, probably due to an increase in the number of CD8+ lymphocytes and Fas-positive T cells, increasing the immune response against the tumor [314]. Experiments with mice performed by different laboratories and using different melatonin concentrations (from 10 to 100 ug/animal or 1 mg/kg PO) have demonstrated the participation of RZR/RORα receptors in the pro-apoptotic effect of melatonin in cancer cells [315,316,317]. In vitro studies with colorectal cancer cell lines (LoVo, CaCo-2, and TP4 cells) also demonstrate the role of melatonin in inducing apoptosis and inhibiting proliferation. On the one hand, melatonin induces the phosphorylation and translocation of the histone deacetylase HDAC4, driving apoptosis in cancer cells [318]. On the other hand, melatonin inhibits the transcription and release of end-1, a survival factor secreted by several solid tumors and involved in proliferation, angiogenesis, and bypassing apoptosis [319].

Undoubtedly, melatonin also exerts an antioxidant function in the context of CRC. Due to its amphiphilic properties, this indolamine can protect cells against lipid peroxidation through the release of peroxyl, hydroxyl radicals, superoxide anions, and peroxynitrite, which occurs in the advanced stages of the disease due to tissue damage [320,321,322].

Although the role of pineal melatonin in the incidence of CRC tumors has been stablished, we cannot ignore that the intestine and the microbiota are great producers of melatonin and its homeostasis is equally important for the proper functioning of the intestinal system. Thus, CRC is a great example of how pineal and extra-pineal melatonin need to work together for a properly organized organ physiology. As brilliantly reviewed in [323], the pineal and extrapineal melatonin secretion dance is not random and should be finely regulated. However, in terms of molecular regulation, much remains to be unraveled. 

## 6. Novel Roles of Melatonin: Microbiota and Epigenetic Regulation

Despite the fact that a myriad of roles have been described for melatonin, different studies point to other functions which have been recently described. Among these novel roles, we will focus on its function in the dynamic balance of the intestinal microbiota and also in epigenetic regulation. Therefore, this section is intended to put the spotlight on other functions of great relevance in cell/organ homeostasis. 

### 6.1. Melatonin, Microbiota and Cancer

The gastrointestinal tract is in intimate relation with the gut microbiota, a complex and dynamic population of microorganisms with an important role in maintaining immune status and metabolic homeostasis in the host while protecting against pathogens. Indeed, dysbiosis (altered gut bacterial composition) is associated with the pathogenesis of different inflammatory diseases, and also is implicated in the development of different types of cancers. Particularly, the role of the microbiota has been demonstrated in the pathogenesis of stomach (influenced by *Helicobacter pylori*), colorectal (*Escherichia coli*, *Fusobacterium* spp. and *Bacteroides fragilis*), and bladder (*Salmonella enterica typhi*) cancers and other neoplasms such as lymphoma, sarcoma, prostate cancer, breast carcinoma, pancreatic cancer, ovarian cancer, and hepatocellular carcinoma (reviewed in [10]). Thus, maintaining the microbiota composition in good shape becomes of great importance to preserve health and well-being.

Although diet is considered one of the main factors to affect the gut microbiota, other conditions may have an impact on the equilibrium between commensal/pathogens gut bacteria. Indeed, sleep deprivation has been demonstrated to disturb the intestinal microbiota while affecting both plasma [324] and gut [325] melatonin levels. Interestingly, supplementation with exogenous melatonin have been shown to restore the composition of the microbiota [8], probably by reducing oxidative stress [324,326] and/or inflammatory response [324,325,327] through TLR4 (Toll-like receptor 4)-associated signaling pathways. Indeed, TLR4 is an important receptor for intestinal microbial response, which also suggests that melatonin can directly interact with the intestinal microbiota [327,328]. This is also supported by the fact that some bacteria in the microbiota express sequences very similar to the melatonin binding sites in MT1 and MT2 receptors [90] and that melatonin can affect the motility and activity of *Enterobacter aerogenes*, a specific human gut bacteria [329]. Melatonin has been also recently found to promote goblet cell differentiation and to induce Reg3β (an antimicrobial peptide against Gram-negative bacteria) in mice. In human intestinal epithelial cells (in vitro), melatonin has been shown to promote mucin and wound healing and to inhibit the growth of *Escherichia coli* [327]. Not only sleep disruption but also constant light may produce dysbiosis in mice [330], with exogenous melatonin restoring this deleterious effect. The microbiota, in addition, might be a source of melatonin [5], which means that melatonin might be also a molecule that serves as a signal for the microbiota to communicate with the host. 

All these findings suggest that melatonin might constitute an important link between sleep deprivation or ALAN and dysbiosis, with the consequent health challenges, cancer included, that can arise from those microbiota alterations. Thus, the maintenance of the microbiota composition might be another important melatonin function that has just started gaining attention.

Other host hormones and neuro-hormones can also modify the microbiota composition—for example, in stress conditions. Gastro-intestinal entero-endocrine cells can secrete over 30 hormones involved in different biological functions (motility, digestion, neuromodulation) that are sensed by enteric bacteria, influencing their composition. This is the case with leptin and ghrelin, which can modulate the gut microbiota composition [331,332]. Conversely, commensal bacteria not only produce vitamins (K and B) or catabolize secondary bile acid, but can also produce hormone-like metabolites, such as short-chain fatty acids (SCFAs), which, once produced, go to the liver to participate in glucose and lipidic metabolism [333,334]. Thus, the gut represents a complex system that is in an intimate relationship with the nervous system through the “gut-brain axis” (GBA), so that the central nervous system, the autonomic nervous system, the enteric nervous system, the hypothalamic-pituitary adrenal axis, and the entero-endocrine system communicate and trigger a response in the gut and vice versa. Therefore, it is easy to understand the importance that the microbiota and its composition have in this bidirectional communication [335].

#### Microbiota and Cancer

Given the complex, relevant, and balanced communication between the microbiota and the host, it is not surprising that the rupture of this balance can lead to pathological processes including cancer. Indeed, the role of the microbiota in cancer is currently a focus of study. Up to date, tumor-suppressor and tumor-promoter activities have been found to depend on the bacteria composition [335]. Several microbial metabolites, including bacterial butyrate and propionate, inhibit cancer cell growth by HDACs inhibition in CRC and lymphoma. Ferrichrome, a metabolite secreted by *Lactobacillus casei*, induces apoptosis in tumor cells through the activation of the JNK pathway [336], while lipopolysaccharide from the outer membrane in Gram-negative bacteria is able to activate host immune response via TLR4, triggering T cell-mediated anticancer activity [337]. Additionally, commensal bacteria and their metabolites can induce IL-18 production, which is essential for the maintenance of the gut barrier. Deficiency in IL-18 production results in an intestinal barrier impairment which causes larger commensal bacteria penetration and increased inflammation, which may eventually trigger tumorigenesis both in the colon and in distal organs such as the liver [338,339]. Therefore, when the gut-microbiota balance gets disrupted and therefore the bacterial composition changes, the role of these new more abundant commensals can favor tumor growth. Among the processes that dysbiosis can induce, we find DNA double-stranded breaks, p53 degradation, β-Catenin, MAPK and AKT pathways activation, antitumor-response blockage, ROS production, and ER activation.

Due to the role of melatonin in regulating the enteric microbial composition, it would be intuitive to suggest the existence of a melatonin-microbiota-cancer axis (MMCA). This regulatory role is primarily attributable to pineal melatonin. However, the fact that both enteric cells and microbiota also produce melatonin cannot be ignored. In fact, these three vertices of the melatonin triangle in the intestine (pineal-enteric cells-microbiota), its balance, and its relationship with GBA and MMCA homeostasis are a fascinating subject in which much remains to be discovered.

### 6.2. Epigenetic Regulation

As previously documented, the actions of melatonin include different molecular and physiological perspectives. With regard to the remarkable pleiotropy of melatonin, it has been found that different levels of gene regulation are exerted by melatonin. Although some melatonin actions in gene regulation are easily explained by its interaction with MT1 and MT2/G protein-coupled receptors, considering the diverse experimental findings there must be other levels of gene regulation exerted by melatonin as an epigenetic factor. While several studies have shown an activation of ERK1/2 by melatonin in an MT receptor-dependent manner, others have demonstrated a suppression of ERK1/2, which is probably explained by other levels of regulation, independent of its receptors [340,341]. Interestingly, the dual, lipophilic, and hydrophilic nature of melatonin enables it to cross the cell and nuclear membranes, reaching organelles and DNA.

Epigenetic modifications are frequent events in the tumor process. Promoter methylation at areas enriched in cytosine and guanine, known as CpG islands, or histone modification (acetylation, methylation, phosphorylation, ubiquitination), have been recorded as frequent events in cancer biology. In this regard, melatonin and its metabolites could have a direct effect on both processes.

DNA methyltransferases (DNMTs), catalyzing CpG island methylation, play a crucial role in epigenetic regulation. According to Korkmaz and Reiter’s hypothesis, both melatonin and its metabolites have similar structures to DNMTs inhibitors, inferring thus a possible role of melatonin in the regulation of DNMTs and therefore of DNA methylation [342]. In support of that, different in vitro and in vivo studies have demonstrated that melatonin could mediate the inhibition of DNA methylation in several genes [343,344] including ARH1 in breast cancer xenograft mouse models [345]. Other studies evidence the fact that melatonin nuclear receptors and their co-regulators are key components in the regulation of certain gene expression through DNMT inactivation [346,347]. In this regard, melatonin treatment is able to downregulate the expression of genes that are under the control of estrogen binding to ER, pointing to an epigenetic control of melatonin in ER [348]. Moreover, it seems that melatonin regulates hTERT expression, commonly upregulated in cancer, via DNMT inhibition in an MT-independent manner [342,349]. 

Another level of epigenetic control is carried out by the post-translational modification of proteins. Among them, the balance between acetylated and deacetylated proteins, mainly histone, plays a very important role in gene expression. In fact, erroneous acetylation by histone acetyl transferases (HATs) enzymes or deacetylation by histone deacetylases (HDACs) enzymes frequently occurs in certain tumors. Interestingly, different studies have reported the effect of melatonin on the up- and down-regulation of HDAC [350,351]. As mentioned previously, melatonin and SIRT1 (a class III HDAC) are closely related. Although melatonin could modulate the activity of SIRT1 by regulating the cell oxidative state, the direct action of the indoleamine on SIRT1 expression is not ruled out and different experimental approaches would be required.

Interestingly, the role of melatonin in the regulation of DNMTs and HDACs is not just tissue-dependent. A very exciting work that assesses the role of melatonin in kidney hypertension and nephrogenesis in rats has demonstrated a differential role of melatonin in epigenetic regulator genes depending on age. In 1-week-old offspring, melatonin up-regulates DNA methyltransferase 3A (*Dnmt3a*), histone deacetylase 4 (*Hdac4*), histone deacetylase 7 (*Hdac7)*, histone deacetylase 1-like (*Hdac1l*), chromodomain helicase DNA binding protein 1 (*Chd1*), *Chd2*, and *Chd3*, among others. However, at 16 weeks of age, melatonin only downregulates *Dnmt3b* and up-regulates *Dnmt3l* and *Hdac4* [352].

## 7. Pineal vs. Extrapineal Melatonin in Cancer

In an attempt to differentiate those functions of pineal from those of the extrapineal melatonin, we could assume that the former has chronobiotic and indirect antioxidant functions, while the latter would present more local effects. Additionally, considering the needed concentrations of the indoleamine to act on different processes, we could infer what type of melatonin, in terms of its origin, is acting on the different pathways. Thus, those pathways that require melatonin in higher concentrations than those reached in the blood (secreted by the pineal gland) as well as those pathways that are able to be targeted at the photophase but not during the scotophase will presumably be decided by the local production of melatonin. 

**Figure 5:**
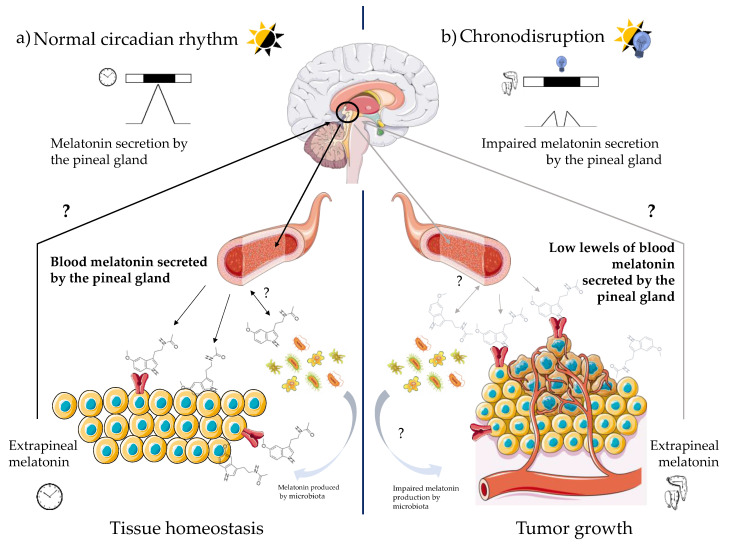
Possible interplay between pineal and extrapineal melatonin (chemical formula in grey) in chronodisruption. (**a**) Situation under normal circadian rhythm. The pineal gland secretes melatonin with a peak during the dark phase, reaching concentrations of 0.5–1 nM in plasma. Its actions may occur in an MT receptor-dependent manner or by diffusion through the plasma membrane. In addition, extrapineal melatonin seems to be in antiphase with melatonin from the pineal gland (concentrations not yet fully stablished). Plasma melatonin may participate in the regulation of extrapineal melatonin production (e.g., melatonin produced by the microbiota or different tissues) and, in turn, extrapineal melatonin may exert some regulation on the pineal melatonin secretion, thus closing a possible feedback-loop. (**b**) Under chronodisruption or ALAN exposure, melatonin secretion is impaired, probably with its total concentration reduced in plasma. When this situation is continued over time, it could affect the production of extrapineal melatonin, driving decreased or mistimed melatonin levels and therefore leading to peripheral clock mismatch and reducing the ability to control tumor growth. Another possibility that remains to be explored is whether the crosstalk between extrapineal and pineal melatonin is affected by chronodisruption, driving a malfunction of the feedback loop and contributing to the further promotion of the tumor. This figure was built with SMART resources (Servier Medical Art), licensed under a Creative Common Attribution 3.0 Generic License. See http://smart.servier.com/.

At this point, we would ask what type of melatonin (pineal or extrapineal) exerts antitumor effects in cancer. As suggested in Figure 5, the secretion and activity of both melatonins are probably tightly regulated. To be able to answer this question, different experimental approaches would be required to distinguish the activity of both origins. We could infer that local melatonin would exert a constant scavenging effect, while pineal melatonin would indirectly and directly act during the night, when levels are detectable. However, great effort will be required to understand the separate roles of melatonin during light and dark phases. In any case, as previously discussed, it is likely that pineal melatonin regulates local melatonin production. Unraveling how this regulation works will be essential to differentiate both effects in cancer.

## 8. Conclusions and Future Perspectives

The fact that melatonin appeared early in evolution and is synthesized by most clades suggests its biological importance. With this article, we aimed to review the range of melatonin features that make it an essential molecule in physiology, particularly in the pathophysiology of cancer. As reviewed, melatonin is an indoleamine that presents antioxidant, chronobiotic, immunomodulatory, antiproliferative, pro-apoptotic, and anti-angiogenic functions, among others, highlighting the importance of this molecule in protection from cancer development. Indeed, there is increasing epidemiological, physiological, and molecular evidence that points to a role of chronodisruption in cancer development, where pineal melatonin would present a central role. However, how pineal melatonin interacts with extrapineal melatonin also becomes of interest both in circadian and cancer fields.

Interestingly, melatonin is also involved in microbiota regulation in relation to health and in epigenetic modification, roles that have been recently described and are in close relation to cancer development. However, much remains to be unraveled regarding different essential aspects of melatonin. Along this review, we have emphasized the complexity of the functions of melatonin, its different origins, and the likely complex relationship and interplay between both pineal and extrapineal melatonin. An interesting example of this interaction is the gut–microbiota–brain axis, with enteric cells, the microbiota, and the brain all producing melatonin under different regulating processes.

Thus, unraveling the complex scenario that emerges from the possible interplay between pineal and extrapineal melatonin, the wide range of functions both exert, and their evolutionary significance creates an exciting challenge. Additionally, considering melatonin as one of the possible bridges between lifestyle and health, this research constitutes a thrilling field to explore. Hopefully, this review will help to provide different perspectives to enrich the whole picture of the melatonin field.

## Figures and Tables

**Table 1:** Summary of the effects of melatonin and its mechanisms of action in some of the hallmarks of cancer. [+] stands for activation and [−] for inhibition.

Hallmarks of Cancer	Melatonin Effect	Mechanisms of Action	References
Oxidant status	Maintenance of genome stability ROS scavenger	[+] Antioxidant enzymes (SOD, GPx, CAT) [+] DNA damage response [+] DNA damage repair Decrease expression of *Sirt*	[130,131,132,133]
Chronodisruption	Circadian synchronization with the light/dark cycle	Transcriptinal control of PER1, CRY1 and BMAL1 [−] Proteasomal cleavage of BMAL1 Decrease expression of *Sirt1*	[134,135,136,137,138,139]
Immune evation, inflammation	Immunomodulatory Anti-inflammatory	Stimuation the release of IL-2, IL-6, IL-12, IFN-γ, TNF-α. Suppression of IL-4 Increment of NK cells and monocytes Enhancement of antigen presentation by macrophages Suppression of eosinophils and Th17 cells Modulation of IL-2/IL-2 receptor system Downregulation of *Foxp3* in Tregs [+] CD4(+) T helper cells	[67,72,140,141,142]
Sustained proliferation	Antiproliferative	[−] Transcription of cyclins and CDKs (Cyclin d1, Cyclin b1, Cdk4, Cdk1) [−] Phosphorylation of PI3K, AKT, PRAS40, GSK-3, and MDM2 When melatonin administered with VitD: [+] TGFβ [−] Phosphorylation of AK Reduction in MDM2 values. [−] Telomerase activity by inhibition of *Tert*	[143,144,145,146,147,148,149,150]
Resistance to apoptosis	Induction of apoptosis Regulation of the homeostasis between apoptosis and autophagy	Decrease BCL2 levels Increase BAX levels Upregulation of p53 Upregulation of cleavage-caspase Downregulation of survivin [−] *Myd88* and *Trif* Enhancement of MST1 [+] Procaspase enzymes via p38 and JNK activation and NF-κB suppression	[151,152,153,154,155,156]
Increased angiogenesis	Angiogenesis inhibition	[−] HIF-1a and VEGF [−] GSK-3β	[157,158]
Metastasis	Cytoskeleton rearrangement Downregulation of anchoring proteins Inhibition of EMT	Upregulation of E-cadherins, occludins, and integrins Reduce expression/activity of MMP-9 and MMP-2 Downregulation of vimentin Downregulation of *Snail* and *Slug* and attenuation of Wnt/β-Catenin pathway	[159,160,161,162,163,164,165]

**Table 2:** Clock genes in different types of cancer.

Type of Cancer	Core Clock Genes Modification in Cancer		Melatonin Function in Cancer		Reference
Breast	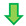	Per1, Per2, Cry2, Bmal1, Rorα	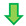	Bmal1, Rorα	[230,232,264,270,271,272,273,274]
	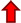	Clock, Rev-Erbβ	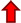	Per2, Cry2	
Prostate	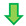	Per1, Per2, Clock	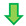	Bmal1	[263,272,275,276,277]
	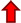	Bmal1, Rorα	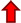	Per2, Clock	
Liver	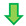	Per1, Per2, Per3, Cry1, Cry2, Clock, Bmal1, Rev-Erbβ	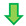	Clock, Bmal1, Cry1, Per1, Per2, Per3, Rev-Erbα, Rev-Erbβ	[263,278,279]
	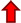	Clock, Bmal1, Rora	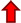	Cry1, Per1, Clock, Bmal1, Rorα, Per2	
Colorectal	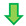	Per1, Per2, Per3, Bmal1	Cry inhibition		[263,280,281,282,283]
	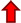	Cry1, Cry2, Bmal1, Rev-Erbα, Timeless.			
